# Ovariotomy in a Girl 12 Years Old

**Published:** 1869-12

**Authors:** 


**Ovariotomy in a Girl Twelve Years Old.**

DR. JOUON, of Nantes, has removed an ovarian tumor, weighing twenty pounds, from a girl only twelve years old, who had never menstruated. A long, narrow pedicle was secured by a clamp, and the patient recovered. The case is reported in the *Gazette Hebdomadaire*. This is probably the youngest patient on whom ovariotomy has been performed, as Mr. Spencer Wells' youngest patient was fourteen. She had not menstruated, and also recovered. Mr. Wells has had three successful cases in girls of seventeen. — *Med. Times and Gaz., Oct.* 9, 1869. *Medical News.*

